# Balancing Breeding for Growth and Fecundity in Radiata Pine (*Pinus radiata* D. Don) Breeding Programme

**DOI:** 10.1111/eva.13164

**Published:** 2020-12-02

**Authors:** Harry X. Wu, Richard Ker, Zhiqiang Chen, Milos Ivkovic

**Affiliations:** ^1^ Beijing Advanced Innovation Centre for Tree Breeding by Molecular Design Beijing Forestry University Beijing China; ^2^ CSIRO National Research Collection Australia Canberra ACT Australia; ^3^ UPSC Swedish University of Agriculture Sciences Umeå Sweden; ^4^ Tree Breeding Australia Mount Gambier SA Australia

**Keywords:** breeding, evolutionary constraint, fecundity, genetic correlation, heredity, radiata pine

## Abstract

Tree breeding has focused on increasing stem volume growth with a cost to fecundity. However, fecundity is important in maintaining the fitness in natural stands and facilitating cross‐pollination to advance breeding populations. Understanding the inheritance of fecundity and the genetic relationship between fecundity and growth is essential to understand the constraints of evolution in natural population and design an optimal selection strategy to balance breeding for growth and fecundity. Inheritance of female fecundity and the genetic relationship between fecundity and growth in radiata pine were investigated using a large Australia‐wide progeny test, planted on eight sites involving 279 control‐pollinated families. It was found that fecundity of female cones was highly heritable with an estimated heritability of 0.39–0.61, but genetically correlated with growth (−0.30 to −0.39). This indicates that improvement in tree growth alone could reduce the fecundity, thus to break the possible evolutionary constraint in natural population. To maintain fecundity for breeding purposes and minimize the interruption of the evolutionary constraint between fecundity and growth, use of a restraint selection index to impose no change of fecundity is developed in current breeding, while dissecting the genetic basis of adversely correlated traits at loci level is required for optimal long‐term strategy.

## 1 INTRODUCTION

Grain yield is a major breeding objective trait in most agronomic crops. Increases in grain yield have been achieved mainly at the expense of stem biomass due to the genetic selection of flowering genes shifting the proportion of vegetative growth to reproductive growth, and the gibberellic acid hormone system preventing plants from collapsing by reducing plant height (Peng et al., 1999; also see review by Eshed and Lippman, 2019). In contrast, tree breeding worldwide has focused on increasing stem biomass. An important question for forest tree geneticists is whether breeding for increased stem volume is achieved at the cost of reduced reproductive fitness, as measured by quantitative traits such as seed production capacity and flowering time onset? Maintaining the fecundity of forest trees is important for breeding operations: facilitating the crossing of parents under recurrent selection; and producing the deployment population (seeds and seedlings for reforestation). Conifer tree improvement programmes typically involve advancing the breeding population via the controlled mating of elite trees selected from field trials, and the production of the planting (deployment) population via the establishment of seed orchards using elite trees with the highest estimated breeding values (EBV).

In terms of tree physiology, trees diverting energy resources to seed production may have less resources available for vegetative growth. The study of four stands of radiata pine (*Pinus radiata*) in Canberra indicated that on average a 16% mean annual increment (MAI) of volume was diverted to cone and pollen production and this is equivalent to a reduction 2.4 m^3^ of wood on an average MAI = 15 m^3^ plantation (Fielding, 1960). Therefore, breeding for reduced cone and pollen production (referred as fecundity in this paper) seems an attractive option to boost productivity.

On the other hand, production of seeds requires maintenance of fecundity. Fecundity of trees is affected by age, environment, genetic composition and interactions among these factors (Kang & Lindgren, 1998; Krannitz & Duralia, 2004). Differences in fecundity were usually higher during poor flowering years and in young populations, and fecundity has been shown to vary considerably within and between populations based on data assembled from 99 stands and 36 seed orchards (Kang et al., 2003). Inbreeding can also reduce or delay fecundity in radiata pine (Wu et al., 2004). Variation in fecundity between individuals has been shown to influence mating patterns in seed orchards, resulting in increased levels of inbreeding in seeds (Kang & Lindgren, 1998) and affecting genetic diversity in crops (Eriksson et al., 1973, El‐Kassaby & Cook, 1994, and Bila, 2000).

Studies assessing genetic variation in fecundity, as measured by differences in cone and seed production, typically use data collected in seed orchards because such traits are difficult to measure in field trials. Such studies have shown that differences in fecundity between clones are partly due to genetic factors (Burczyk & Chalupka, 1997; Byram et al., 1986, Kjer & Wellendorf, 1998). In ponderosa pine, prolific cone‐producers as a group have been shown to be markedly different from low cone‐producers at three protein loci (Linhart et al., 1979).

Clonal repeatabilities (individual‐tree broad‐sense heritabilities) between 0.30 and 0.64 for fecundity traits have been estimated from clonal seed orchards in radiata pine (Fielding, 1960, Burdon & Low, 1973 and Griffin, 1982). Similar clonal repeatabilities between 0.38 and 0.40 in a Douglas‐fir orchard and between 0.34 and 0.41 in a Sitka spruce orchard have been reported (Chaisurisri & El‐Kassaby, 1993; El‐Kassaby & Cook, 1994). A higher clonal repeatability was about 0.51 in a loblolly pine seed orchard (Schmidtling, 1983).

While fecundity variation has been a major concern in seed orchard management, it is typically not included as either a breeding objective trait or selection criteria in tree breeding programmes, particularly in conifers, due to practical difficulty in measuring the trait. However, reproductive traits are expected to decline if they are not directly selected, have genetic variance and are unfavourably correlated with traits under strong selection. There are few documented studies using well‐designed genetics trials that document narrow‐sense heritability for fecundity in conifers. Individual‐tree and family‐based heritabilities of 0.55 and 0.77, respectively, were reported in a non‐commercial Ocala sand pine (*Pinus clausa*), in Florida (Lockwood & Goddard, 1980). In slash pine (*Pinus elliottii*), a clonal repeatability of 0.50 and an individual‐tree heritability of 0.13 were observed for fecundity (Varnell et al., 1967). Schmidtling (1983) estimated an individual‐tree heritability of 0.61 for the average number of flowers in a diallel trial of 10 parents in loblolly pine (*Pinus taeda*). High family heritability and genetic gain for cone and seed production were also observed in a combined provenance and family trial of *P. tecunumanii* (Nyoka & Tongoona, 2001).

As conifer tree breeding populations advance to the fourth and higher generations with strong selection pressure for growth, form, and other traits (Isik & McKeand, 2019; Kerr et al., 2015; Wu et al., 2007), the effect of such selection on fecundity remains largely unknown. Hence obtaining genetic correlations between growth and fecundity traits will be important for quantifying whether traditional selection methods without considering fecundity has had an adverse effect on fecundity in the selected population. These knowledges are essential to understanding the evolution of correlated traits. On a more practical level this understanding is also needed in order to design the optimal selection strategy for balancing growth and fecundity in breeding.

**Figure 1:**
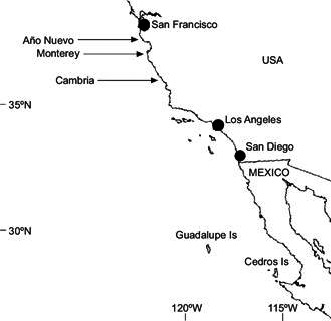
Five native populations of radiata pine in USA (Ponit Año Nuevo, Monterey, and Cambria) and Mexico (Guadalupe and Cedros Islands)

The radiata pine breeding programme entered the third generation in Australia about a decade ago (Wu et al., 2007). Selection was based on growth and form traits for the plus‐tree selection phase in the first generation of breeding (Wu & Matheson, 2002) and on an economic index comprising wood stiffness and stem straightness in addition to growth and form, in the third and ensuing generations (Kerr et al., 2015; Wu et al., 2008). Anecdotally, it was observed that radiata pine trees varied in their ability to produce male and female flowers and cones in seed orchards. In progeny trials, some of the faster‐growing trees had delayed flowering and had fewer flowers in the second and third generation (Wu et al., 2004). Radiata pine is a native species in North America with only five native populations left in California and two Mexico islands (Figure 1). The total land area inhabited by native populations has been reduced to about 4,500 hectares due to climate change, urbanization and new diseases. The species is listed as a threatened species by an international union for conservation of nature (IUCN) (Farjon, 2013). A significant effort of in situ and ex situ conservation has been implemented (Burdon et al., 2017; Roger, 2002). About 100 conservation stands in Australia and New Zealand from cone collections of the five native populations currently serve as ex situ conservation. There are approximately four and half million hectares of commercial forests planted outside native provenances (Eldridge, 1997). The objectives of this research were to examine the genetic and non‐genetic control (additive, non‐additive, and possible site effects) of fecundity, and in particular to estimate the genetic correlation between the fecundity and growth. The data for this research had been collected from a research working group series of trials which had a deliberate focus on the measurement of fecundity in radiata pine. The possible impact of recurrent selection schemes without consideration of fecundity at the population level has also been addressed for conservation and commercial production.

## 2 MATERIALS AND METHODS

### 2.1 Experiment

**Figure 2:**
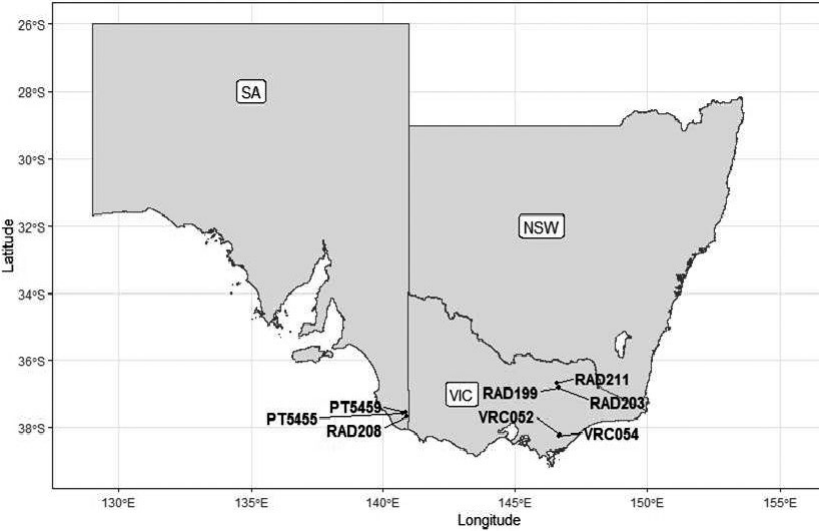
Distribution of eight AWD sites in south‐eastern Australia (NSW—New South Wales, VIC—Victoria, and SA—South Australia)

A total of 124 parent trees from Australia (115 trees) and New Zealand (nine trees) was used in the Australian national mating and testing programme between 1976 and 1987. Trials in this period are referred to as the Australia‐Wide‐Diallel series (AWD). The AWD trials used a series (21 sets) of six‐parent disconnected half‐diallels as the mating design with 15 possible crosses for each set (Griffin, 1976). Eight tests planted in 1986 and in 1987 were assessed for female cone number for this study. Three tests (RAD199, RAD211, and RAD203) were planted in the Murray Valley region of high rainfall (1100–1200 mm) in north east Victoria, and other five tests were planted in south east Victoria and South Australia (PT5455, PT5459, RAD208, VCR052 VCR054) with intermediate rainfall (680–785 mm) (Table 1, Figure 2). Ninety‐eight full‐sib families from 50 parents were planted on three sites (PT5455, VRC052 and RAD199) and 214 full‐sib families from 96 parents were planted on two sites (PT5459 and RAD211) (see detailed design in Wu & Matheson, 2004). Trees from 48 families, derived from 45 parents were planted on the VRC054 site, whereas the remaining two sites (RAD203 and RAD208) had trees from 35 and 23 families, respectively.

**Table 1:** Site characteristics, experimental designs, measured traits (diameter at breast height (DBH), average number of cones per tree, and percentage trees with cones in eight sites of the Australia‐wide radiata pine diallel experiment (AWD)

Site	PT5455	PT5459	VRC052	VRC054	RAD199	RAD211	RAD203	RAD208
State	SA	SA	VIC	VIC	VIC	VIC	VIC	VIC
Year of planting	1986	1987	1986	1986	1986	1987	1986	1986
Latitude (S)	37^o^34’	37 ^o^33’	38 ^o^14’	38^o^15’	36 ^o^50'	36 ^o^41	36 ^o^50'	37^o^40'
Longitude (E)	140 ^o^45’	140 ^o^53’	146 ^o^41’	146° 39’	146 ^o^39'	146 ^o^34’	146 ^o^39'	140^o^54'
Elevation (m)	65	70	93	184	320	370	320	70
Mean annual rainfall (mm)	690	680	760	785	1,200	1,100	1,200	710
Mean Temp. (°C)^b^	13.9	14.0	13.3	13.3	13.5	12.7	13.5	13.9
Soil type	Sandy	Sandy	Sandy loam	Sandy loam	Gravel loam	Sandy loam	Gravel loam	Sandy
Site type^a^	2nd PR	2nd PR	Pasture	2nd PR	Pasture	2nd PR	Pasture	Pasture
Number of parents	50	96	50	45	50	96	18	10
Number of family	98	214	98	48	98	214	35	23
Number of replicates	4	3	4	20	4	3	4	4
Tree per plot	4	4	4	1	4	4	5	5
Spacing (m)	3 × 3	3 × 3	2.8 × 3.6	2.3 × 3.6	3 × 3	3 × 3	3 × 3	3 × 3
DBH (mm)	203 ± 26	161 ± 24	227 ± 23	226 ± 32	223 ± 36	159 ± 33	226 ± 40	198 ± 28
Number of cones per tree	10.8 ± 10.2	7.2 ± 6.7	10.0 ± 9.9	6.4 ± 7.6	6.4 ± 10.2	2.6 ± 4.7	8.7 ± 10.9	17.1 ± 13.1
% Trees with cones	92.4%	88.0%	90.9%	84.9%	64.3%	50.8%	73.9%	97.2%
Age at the assessment (year:month)	9:11	8:06	9:11	10:00	9:10	8:10	10:00	9:07

^a^ 2nd PR—second rotation radiata pine sites, Pasture— ex‐pasture crop sites. Mean Annual Temperature Search Results.

^b^ The mean temperature refers to the long‐term (1960–2012) average of daily min and max temperatures.

All eight tests were located on typical radiata pine plantation sites, from sandy to gravel loam soil. Trees were planted in two types of site*, viz* second rotation radiata pine sites (2nd PR) and ex‐pasture crop sites (Pasture). Ex‐pasture sites were usually more fertile than the second rotation sites due to pasture improvement. The experiments were planted in 3–4 replicates with 4–5 tree row plots except for one site (VRC054) in which single tree plots were used. One‐ and two‐year‐old green cones and brown cones were counted from ground level for all the trees (total 10,195 trees) at a similar age of trials (8 year 6 months to 10 years) (Table 1). All cones were added together to represent the female reproductive ability, referred as fecundity in this paper.

The distribution of total cone numbers for all eight sites was examined, and a skewed distribution towards zero was observed. Consequently, a square‐root transformation was applied to the total cone number. After transformation, five sites (PT5455, PT5459, VRC052, VRC054 and RAD208) had a normal or an approximate normal distribution, whereas the transformed scores at three sites (RAD199, RAD211 and RAD203) were still largely skewed towards zero. These three sites had a higher percentage of trees (>25%) that were not producing any cones. Consequently two additional analyses were conducted. A binomial analysis, treating fecundity as a threshold trait (0 for trees with no cone and 1 for trees with cones, (called fecundity score) were conducted for the three sites (Dempster & Lerner, 1950). An analysis using the percentage of trees bearing cones was also implemented for the RAD199 and RAD211 sites that had the lowest number of trees producing cones (64.3% and 50.8%, respectively).

### 2.2 Statistical model used

The following series of statistical analyses were completed for square‐root transformed total cone number (CONE) and diameter at breast height (DBH) data:

1. Univariate and bivariate single‐site analyses, and across‐sites bivariate analyses were conducted using CONE and DBH, for all eight sites.
2. Binomial analyses where CONE was treated as a threshold trait (fecundity score) for RAD199, RAD211 and RAD203.
3. Percentage of trees bearing cones was analysed after arcsine square‐root transformation for RAD199 and RAD211. The same model for square‐root transformed total cone number was used for the arcsine square‐root transformation percentage.

The second method used to model the data circumvents the requirement for the normal distribution of the data. All analyses were undertaken using the ASReml program (Gilmour et al., 2009).

### 2.3 Individual site univariate analyses

The following mixed linear model was used for individual sites in order to estimate basic genetic parameters for use in further analyses of bivariate and joint‐site analyses.

$$Y=X\beta +Z_{g}\mathrm{GCA}+Z_{s}SCA+e.$$

where **β**, **GCA**, **SCA** and **e** are fixed replicate, random general and specific combining abilities, and residual effects, respectively, **X**, **Z_g_** and **Z_s_** are design matrices corresponding to these effects. The random effects have following distributions:

$$\mathrm{GCA}∼\left(0,G_{\mathrm{GCA}}=\sigma_{\mathrm{gca}}^{2}I\right),\mathrm{SCA}∼(0,G_{\mathrm{SCA}}=\sigma_{\mathrm{sca}}^{2}I\text{and}e∼\left(0,R\right).$$

where $\sigma_{\mathrm{gca}}^{2},\;\text{and}\;\sigma_{\mathrm{sca}}^{2}$ are GCA and SCA variances, **R** is the diagonal residual variance matrix, and I is an identity matrix with dimension as number of corresponding effects. The **Z**_g_ is a special incidence matric formulated for a diallel mating structure. Preliminary analyses indicate there was no significant interaction between GCA, SCA and replication, therefore, these interactions were dropped in the final analyses for the individual and combined sites.

### 2.4 Individual site bivariate analyses

Due to heterogeneous error and GCA variances among sites, we conducted bivariate (DBH and CONE) analyses site by site to estimate genetic correlation between DBH and CONE. For individual site bivariate analyses, the mixed linear model was formulated as.

$$Y=\left[\begin{array}{c} y_{\mathrm{DBH}}\\ y_{\mathrm{Cone}} \end{array}\right],\;\mathrm{GCA}=\left[\begin{array}{c} GCA_{\mathrm{DBH}}\\ GCA_{\mathrm{Cone}} \end{array}\right],\;...,\;e=\left[\begin{array}{c} e_{\mathrm{DBH}}\\ e_{\mathrm{Cone}} \end{array}\right],$$

where *GCA_DBH_, GCA_CONE_* are GCA effect vectors for DBH and CONE, respectively. The incidence matrix, **Z**_g_ becomes.

$Z_{g}=\left[\begin{array}{c} Z_{\mathrm{gDBH}} \end{array}\begin{array}{c} Z_{\mathrm{gCone}} \end{array}\right]=\sum_{⊕}Z_{i}.$

The variance–covariance structures for GCA and residuals become:

$$G_{\mathrm{GCA}}=G_{GCA_{DBH*Cone}}⊗I\;\text{and}\;R=\left[\begin{array}{cc} \sigma_{e_{\mathrm{DBH}}}^{2}I_{1} & \sigma_{e_{DBH*Cone}}I_{12}\\ \sigma_{e_{DBH*Cone}}I_{12} & \sigma_{e_{\mathrm{Cone}}}^{2}I_{1} \end{array}\right].$$

where $G_{GCA_{DBH*Cone}}$ is GCA variance and covariance matrix among DBH and CONE within single site, $\sigma_{e_{\mathrm{DBH}}}^{2}$ is the residual variance for trait DBH and $\sigma_{e_{DBH*Cone}}^{2}$ is the residual covariance between DBH and CONE. Genetic variance‐covariance matrices for SCA effects were estimated in a similar fashion as for GCA.

### 2.5 Across‐sites univariate analyses

An eight‐site model was conducted to test whether there was: (1) a single common versus heterogeneous error variances among sites; (2) a uniform (same) genetic correlation among sites; and (3) a single common variance either for GCA or SCA effects versus heterogeneous GCA and SCA variances. The statistical model used is similar to the model for individual site of univariate analyses, but included site effects. Results from across‐sites univariate analyses were used as initial values for the across‐sites bivariate analyses.

### 2.6 Across‐sites bivariate analyses

We also performed a multiple‐trait, multiple‐sites analysis to estimate between‐trait genetic correlations, accommodating among‐site heterogeneous variances for residual, GCA and SCA, for both traits. The model used to estimate bivariate DBH and CONE correlation for combined sites is.

$$Y=\left[\begin{array}{c} y_{S_{1}DBH}\\ y_{S_{1}Cone}\\ y_{S_{2}DBH}\\ y_{S_{2}Cone}\\ ...\\ ... \end{array}\right],\;\mathrm{GCA}=\left[\begin{array}{c} GCA_{S_{1}DBH}\\ GCA_{S_{1}Cone}\\ GCA_{S_{2}DBH}\\ GCA_{S_{2}Cone}\\ ...\\ ... \end{array}\right],\;...,\;e=\left[\begin{array}{c} e_{S_{1}DBH}\\ e_{S_{1}Cone}\\ e_{S_{2}DBH}\\ e_{S_{2}Cone}\\ ...\\ ... \end{array}\right],$$

Where $y_{S_{1}DBH}\;\text{and}\;y_{S_{1}Cone}$ are vectors containing phenotypic observations for DBH and CONE at site 1 etc. The incidence matrix relating observations to GCA is similarly partitioned as.

$Z_{g}=\left[\begin{array}{ccc} Z_{S1DBH} & 0 & \\ 0 & Z_{S1Cone} & \\ & & \ddots \end{array}\right]=\sum_{⊕}Z_{i}.$

The variance–covariance structure for GCA is.

$$G_{\mathrm{GCA}}=\left[\begin{array}{ccc} G_{01}⊗I_{1} & G_{12}⊗I_{1^{\prime }2} & ...\\ G_{12}⊗I_{1^{\prime }1} & G_{02}⊗I_{2} & ...\\ ... & ... & ... \end{array}\right].$$

where **G**_01_ is the between‐trait GCA variance and covariance matrix within‐site 1 and **G**_12_ is GCA covariance matrix between DBH and CONE between sites 1 and 2 which has elements:

$$G_{12}=\left[\begin{array}{cc} \sigma_{gca_{\mathrm{DBH}}12}^{2} & \sigma_{gca_{DBH*Cone}12}^{2}\\ \sigma_{gca_{DBH*Cone}12}^{2} & \sigma_{gca_{\mathrm{Cone}}12}^{2} \end{array}\right].$$

where $\sigma_{gca_{\mathrm{DBH}}12}^{2}$ is an inter‐site covariance for DBH (sites 1 and 2) and $\sigma_{gca_{DBH*Cone}12}^{2}$ is an inter‐site, inter‐trait. The variance–covariance structure for SCA effects was formulated in the same way. Finally, the residual variance–covariance structure was formulated as.

$$R=\left[\begin{array}{ccc} R_{1} & 0 & ...\\ 0 & R_{2} & ...\\ .. & ... & ... \end{array}\right].$$

where.

$\;R_{1}=\left[\begin{array}{cc} \sigma_{e_{1_{\mathrm{DBH}}}}^{2}I_{1_{\mathrm{DBH}}} & \sigma_{e_{1_{DBH.Cone}}}I_{1_{DBH.Cone}}\\ \sigma_{e_{1_{DBH.Cone}}}I_{1_{DBH.Cone}} & \sigma_{e_{1_{\mathrm{Cone}}}}^{2}I_{1_{\mathrm{Cone}}} \end{array}\right]$.

Models that were able to converge were those assuming a uniform between‐site genetic correlation at the level of GCA effects. These correlations were 0.6 and 0.9 for DBH and CONE, respectively and were applied with and without restraining the ratio of GCA variance to SCA variance.

The combined site analyses were completed for all eight sites and for five sites (PT5455, PT5459, VRC052, VRC054, and RAD208) having a normal or nearly normal distribution of the transformed total cone number.

Narrow‐ and broad‐sense heritabilities were estimated for individual and combined sites. Individual narrow‐sense heritability was calculated, assuming GCA variance estimates a quarter of the additive variance with a coefficient of relatedness of 0.25 among progenies of a single full‐sib family as.

$$h^{2}=\frac{4\cdot \sigma_{\mathrm{gca}}^{2}}{2\cdot \sigma_{\mathrm{gca}}^{2}+\sigma_{\mathrm{sca}}^{2}+\sigma_{e}^{2}}$$

for the individual site analysis and,

$$h^{2}=\frac{4\cdot \sigma_{\mathrm{gca}}^{2}}{2\cdot \sigma_{\mathrm{gca}}^{2}+\sigma_{\mathrm{sca}}^{2}+2\cdot \sigma_{gca.site}^{2}+\sigma_{sca.site}^{2}+\sigma_{e}^{2}}$$

for the combined sites analysis, and where σ^2^_gca_ is the estimate of GCA variance component, σ^2^_sca_ is the estimate of SCA variance component, σ^2^_gca.site_ is the estimate of GCA by site interaction variance, σ^2^_sca.site_ is the estimate of SCA by site interaction variance, and σ^2^_e_ is the residual variance estimate. Standard error of heritability was estimated by a Taylor's series expansion (Stuart & Ord, 1987).

Heritability for threshold trait was estimated according to

$$h_{o}^{2}=h^{2}\;\left\{\frac{{\left[p\left(x_{p}\right)\right]}^{2}}{\Phi_{p}\left(1‐\Phi_{p}\right)}\right\}$$

where p(*x*_p_) is the height of a standard normal curve at the truncation point *x*_p_ and Φ_p_ is the proportion of the population incidence of fecundity. The h^2^_o_ is the estimated heritability of observable fecundity score, h^2^ is the underlying heritability of the unobservable liability (Lynch & Walsh, 1998).

Additive genetic correlations between DBH and CONE were estimated using pooled CONE data of the five sites having normal and an approximate normal distribution and estimated as

$$\frac{\text{cov}(g_{i},g_{j})}{\sqrt{\sigma_{\mathrm{gi}}^{2}\sigma_{\mathrm{gj}}^{2}}}$$

where cov (g*_i_, g_j_*) is the additive genetic covariance between DBH and CONE, and *σ^2^_gi_ and σ^2^_gj_* are the GCA variances for DBH and CONE, respectively.

### 2.7 Selection effect on fecundity

The Australian breeding programme for radiata pine is based on a selection index which economically weights mean annual increment (MAI), wood stiffness, branch index (BIX) and stem sweep; the index does not consider fecundity (Ivković et al., 2006; Wu et al., 2007). The effect of indirect selection on fecundity due to direct selection on DBH (as a proxy for MAI) was investigated in this study. An alternative strategy of maintaining a constant fecundity level, while carrying out selection for DBH was also investigated using a restricted index approach. We used a parametric approach to calculate two selection scenarios as:

(A) selection based on DBH only, but calculating the indirect effect on fecundity.

The direct and correlated responses were calculated as:

$$\Delta G={\;\text{i h}}_{i}^{2}\sigma_{\text{pX}}$$

$$CR_{y}=ih_{x}h_{y}r_{g}\sigma_{\mathrm{PY}}$$

where character X is selected directly and then Y is a correlated character selected indirectly.

(B) index selection based on DBH with the restriction of no change on fecundity.

For restricted selection index, the index equation is

$$I={\;b}_{1}P_{1}+{\;b}_{2}P_{2}$$

where $P_{1}$ and $P_{2}$ are phenotypic measurements of DBH and fecundity on which selection is to be based, and $b_{1}$ to $b_{2}$ are the corresponding weighting factors of the vector to be determined. The b vector derived is (Mrode & Thompson, 2014).

$$b^{*}={\left[\begin{array}{cc} P^{*} & G^{*}\\ G^{*} & 0 \end{array}\right]}^{‐1}\times \left[\begin{array}{c} G^{**}\\ 0 \end{array}\right]\times a$$

where P* is the phenotypic variance for selection trait of DBH, G* is the additive genetic variance–covariance between DBH and fecundity, G** is the additive genetic variance–covariance matrix between selected traits excluding restricted traits, **0** is a vector of zeros, and **a** is the vector of economic weights. By varying the relative economic weights of **a** ($a_{1}$ and $a_{2}$), the genetic responses (gain or loss) for DBH and CONE were also modelled and plotted.

## 3 RESULTS AND DISCUSSION

### 3.1 *Rainfall*versus*cone production*

Among the eight sites surveyed, the average number of cones ranged from 2.6 (RAD211) to 17.1 cones per tree (RAD208) (Table 1). RAD211 also had the lowest percentage of trees producing cones (50.8%), whereas RAD208 had the highest percentage of trees bearing cones (97.2%). This caused a high correlation between the number of cones per tree and the percentage of trees bearing cones among the sites (r = 0.79, *p* < .05). Dickson et al. (2000) report temperature and rainfall had the most impact on cone number in sites surveyed in New Zealand. We have found that rainfall seems to have a large effect on female fecundity in sites surveyed, but did not observe a relationship between the temperature and fecundity. Three sites from the relatively higher rainfall region (north east Victoria with an annual rainfall between 1,100 and 1200mm) had the lowest percentage of trees producing cones at age 9–10 years (no cones produced in 26.1%, 35.7% and 49.2% of trees in RAD203, RAD199, and RAD211, respectively). The other five sites in the southeast of South Australia (PT5455, PT5459, RAD208) and southern Victoria (VRC052 and VRC054) with relatively lower annual rainfall (from 680 to 785 mm) had an average of 90.7% trees producing cones. The high rainfall sites were associated with high elevation sites in this study and their Pearson moment correlation was 0.95 (*p* < .05). Thus, it seems that regions of lower and middle rainfall and lower elevation, such as southern Victoria and South Australia, are synonymous with higher fecundity rates at earlier ages. We did not observe a relationship between site type (viz. a 2nd rotation or ex‐pasture sites) with cone setting in these eight sites. To model the effect of site related climate and soil on cone setting, a more comprehensive sampling of sites with varying factors is recommended.

Other studies have reported that site related factors influence cone setting in radiata pine. Sweet (1975) reported a more than 10‐fold variation in cone number across open‐grown radiata pine stands. Burdon and Low (1973) observed that a phosphate‐deficient site produced lower numbers of cones and seeds.

## 4 CONE NUMBER HAS HIGHER HERITABILITY, LOWER NON‐ADDITIVE GENETIC VARIANCE AND GXE INTERACTION THAN GROWTH TRAIT (DBH)

### 4.1 Single‐site analyses

Pine trees produce both male and female cones (strobili). Female cones (ovulating cones) develop two ovules on its upper side of each cone scale and accept pollination in the spring. Fertilization and seed development are a long process and take up to two years after pollination. Therefore, both first‐year cones and second‐year cones are usually co‐existing on trees and can be easily recognized in radiata pine. Both the age of onset of reproduction (precocity) and abundance of flowers (and cones) each year affect fecundity of plants (Kang & Lindgren, 1998). At the eight sites studied, these two traits may be correlated but remain confounded. No attempt had been made to record the time of the first flowering (e.g. onset of reproduction). Nevertheless, the variation in percentage of trees harbouring strobili were examined at the two sites with the lowest reproductive ability (RAD199 and RAD211) and may give us an indication of the genetic variability in precocity. At the age of nine years, six families had not started to produce any cones at RAD211, whereas nine families had all trees (100%) producing cones. Similarly, seven families in RAD199 had all trees producing cones, whereas three families had only one or two trees out of a total of 16 trees in each family producing cones at the age 10. The narrow‐sense heritability for the percentage of trees flowering was estimated as 0.72 ± 0.13 and 0.35 ± 0.11 for RAD199 and RAD211, respectively.

The genetic analyses of CONE show that GCA was significant for all eight sites at 1% level, whereas for DBH, GCA was significant for only five sites (Table 2). SCA was significant at four sites each for DBH and CONE, respectively. However, the average SCA/GCA variance ratio was much higher for DBH (274% relative to 30% for CONE). Higher SCA/GCA variance ratio in DBH was consistent with a previous study which showed that non‐additive genetic variance was an important component of the phenotypic variance for DBH in this radiata pine population (Wu & Matheson, 2005).

**Table 2:** Estimated variance components for general combining ability (GCA) and specific combining ability (SCA), narrow‐ and broad‐sense heritabilities for diameter at breast height (DBH) and number of cones, genetic correlation between DBH and number of cones for the eight individual radiata pine AWD sites (number of cones was square‐root transformed)

**DBH**								
	Sites							
Sources of variation	PT5455	PT5459	VRC052	VRC054	RAD199	RAD211	RAD203	RAD208
GCA	11.9	8.5*	31.1**	11.1	47.3*	13.4	139.6**	95.6**
SCA	78.5**	24.6*	3.1	71.0**	22.9	84.3**	24.9	46.1
esidual	542.7	376.8	472.5	893.8	1,146.1	949.9	1,277.9	647.7
SCA/GCA (%)	657	290	10	640	48	631	18	48
SCA/(2*GCA + SCA)(%)	77	59	5	76	19	76	8	19
Narrow‐sense heritability	0.07 ± 0.04	0.08 ± 0.03	0.23 ± 0.07	0.05 ± 0.04	0.15 ± 0.06	0.05 ± 0.04	0.35 ± 0.13	0.43 ± 0.17
Broad‐sense heritability	0.56	0.32	0.25	0.33	0.22	0.37	0.42	0.64

Number of Cone								
	Sites							
Sources of variation	PT5455	PT5459	VRC052	VRC054	RAD199	RAD211	RAD203	RAD208
GCA	0.285**	0.183**	0.313**	0.226**	0.442**	0.145**	0.550**	0.261**
SCA	0.118*	0.005	0.060*	0.129	0.100*	0.073*	0.057	0.078
Residual	1.594	1.347	1.722	1.414	2.263	1.138	2.430	2.073
SCA/GCA (%)	41	3	19	57	23	50	10	30
SCA/(2*GCA + SCA)(%)	17	1	9	22	10	20	5	13
Narrow‐sense heritability	0.50 ± 0.15	0.43 ± 0.11	0.52 ± 0.13	0.45 ± 0.15	0.55 ± 0.19	0.39 ± 0.18	0.61 ± 0.23	0.39 ± 0.18
Broad‐sense heritability	0.71	0.44	0.62	0.71	0.67	0.58	0.68	0.51
Genetic correlation	−0.35 ± 0.17	−0.12 ± 0.18	−0.51 ± 0.15	−0.82 ± 0.88	0.06 ± 0.21	−0.40 ± 0.28	0.50 ± 0.24	0.08 ± 0.45

* , **—significant level at 5% and 1%, respectively.

Narrow‐sense heritability for CONE varied from 0.39 to 0.61 for individual sites with an average of 0.48, whereas broad‐sense heritability ranged from 0.44 to 0.71 with an average of 0.61 (Table 2). These heritabilities for CONE were higher than the heritabilities for DBH (*viz* from 0 to 0.42 with an average of 0.17 for narrow‐sense heritability and from 0.22 to 0.66 with an average of 0.40 for broad‐sense heritability estimate, Table 2).

Treating total cone number as a threshold trait for the three sites (RAD199, RAD203, and RAD211) produced a similar estimate of heritability for RAD199 and RAD211 (0.50 and 0.43 respectively). However, the estimated heritability for RAD203 was outside the bounds of the parameter space (>1.0). The higher cone incidence (73.9%) at RAD203 and very high observable binomial heritability estimate (h^2^_o_ = 0.95) may contribute this extreme estimate for RAD203.

### 4.2 Across‐sites analyses

Analyses of the combined eight sites revealed that both GCA and SCA were significant for CONE and DBH, and furthermore there was significant GxE at the level of GCA and SCA effects. For CONE, the best‐fitting model contained a uniform correlation among sites and heterogeneous variances across sites. For DBH, the best‐fitting model contained a uniform correlation and a uniform variance among sites.

Detailed results using compound symmetry models, for only the five sites with normally distributed data, are presented. A significant SCA by site effect and a non‐significant GCA by site effect were observed for CONE (Table 3). In contrast, both GCA by site and SCA by site interactions were significant for DBH. The variance ratio of GxE relative to G is small for the CONE (16% and 19% for GCA and SCA, respectively) in contrast to the very large variance ratio for DBH (173% and 125% for GCA and SCA, respectively). The GxE patterns were also revealed in the across‐sites analyses by estimating between‐site genetic correlations for GCA and SCA. There were very high among‐site genetic correlations of 0.91 and 0.72 for GCA and SCA, respectively, for CONE indicating non‐significant and small GxE for CONE. Moderate among‐site genetic correlations of 0.55 and 0.57 for GCA and SCA, respectively for DBH confirm large and significant GxE for DBH. The large and significant GxE for DBH was consistent with other reported studies for radiata pine (Wu & Matheson, 2004, 2005). As indicated by Shelbourne (1972), only an interaction term with a variance that is more than 50% of the total genetic variance has practical importance for selection and breeding programmes. Therefore, further examination of GxE in CONE was not undertaken. Interestingly, SCA variance was only 14% relative to GCA variance for CONE whereas SCA was 155% relative to GCA variance for DBH.

**Table 3:** Estimated variance components for general combining ability (GCA), specific combining ability (SCA), narrow‐ and broad‐sense heritabilities and genetic correlations for diameter at breast height (DBH) and number of cones in the combined five radiata pine AWD sites (number of cones was square‐root transformed)

Sources of variation	DBH	Number of Cones
GCA	10.498**	0.210**
SCA	16.251**	0.029**
GCA × Site	18.191**	0.033
SCA × Site	4.102*	0.005*
Residual	523.208	1.574
SCA/GCA (%)	155	14
SCA/(2*GCA + SCA)(%)	44	6
Narrow‐sense heritability	0.07 + 0.02	0.41 + 0.05
Broad‐sense heritability	0.18 + 0.04	0.46 + 0.05
Additive genetic correlation	(−0.39 + 0.16)	
Total genetic correlation	(−0.30 + 0.11)	

* , **indicate significant level at 5% and 1%, respectively.

Analyses of variance based on individual and combined sites indicate that CONE had a smaller ratio of SCA and GxE variance relative to GCA variance than in DBH. Heritability for CONE was also much higher than DBH. This may indicate it could be easier to locate alleles with relatively large effects in fecundity than for growth traits such as DBH (Hall et al., 2016). Indeed, major alleles were observed for fecundity related traits such as flowering time and floral meristem formation in trees (Bohlenius et al., 2006; Hsu et al., 2011; Tylewicz et al., 2015). AGAMOUS (AG) subgroup of MADS‐box genes and Leafy (LFY) genes are two examples (Klocko et al., 2020). For DBH, more numerous genes with small effects have been estimated in trees (Hall et al., 2016). In view of the possible larger allele effect sizes and higher heritability for fecundity traits, selective breeding for fecundity alone may be more effective than for a growth trait such as DBH.

## 5 SIGNIFICANT NEGATIVE GENETIC CORRELATION BETWEEN FECUNDITY AND THE GROWTH TRAIT DBH

**Figure 3:**
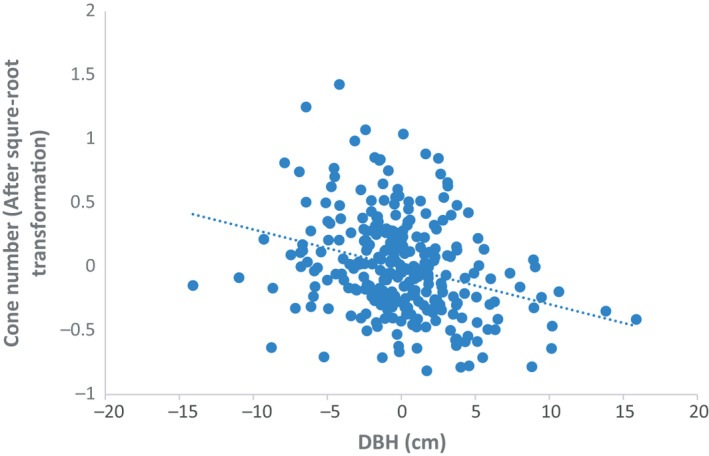
Scatter plot of parental breeding values between the CONE and DBH in the combined five sites

The additive genetic correlation between DBH and CONE was estimated by a bivariate model within individual site analyses and by using a bivariate model within combined five‐site and eight‐site analyses. Estimates from individual site analyses varied greatly and indicate that the estimates were not very reliable due to small sample size at several sites (*viz* VRC052, RAD203 and RAD208, Table 2). Estimated genetic correlation was −0.39 (Table 3) based on a combined five site analysis with normally distributed data without accommodating heterogeneous genetic and error variances. The estimated correlation was −0.29 based on the same five sites and −0.21 based on all eight sites accommodating heterogeneous GCA, SCA and error variances among sites. We also plotted the relationship between breeding values of DBH and CONE, for parents only, across the combined five sites with normally distributed data (Figure 3). A negative genetic correlation can be observed in the scatter plot. There were no obvious points that indicate individuals with a potential to reverse the trend. Log‐likelihood ratio tests confirmed all negative correlations were significantly different from 0. We also estimated and environmental correlation (0.19) and phenotypic correlation (0.01) between the CONE and DBH.

The study of genetic correlations is important because they help define the genetic architecture of a species. The definition of a large inter‐trait correlation matrix is central to the genetic analysis of a species‐, or program‐wide database containing field‐based data, genomic data and pedigree. Such an analysis will generate genetic values that provide the basis on which to make selection decisions for a national, or species‐wide breeding programme. Genetic correlations affect the multi‐trait responses to index selection, and also the correlated responses of secondary traits including fitness traits not included in the index (Lynch & Walsh, 1998).

Recently, biologists have become increasingly cognizant of how traits evolve in a correlated fashion and how the concept of trait trade‐offs underpins much of the research in evolutionary ecology (Conner, 2002; Futuyma, 2010). It is generally believed that a genetic correlation is chiefly due to pleiotropy and that linkage disequilibrium is a cause only of a transient correlation (Falconer & Mackay, 1996). Among evolutionary biologists, pleiotropy is usually regarded as a constraint in evolution in the sense that the correlated response that it causes is deleterious, thereby constraining the primary trait from evolving (Arnold, 1992). Forest trees are in the early generations of domestication with large effective population sizes, are typically outcrossing and have low LD observed (Neale & Kremer, 2011). Both LD and pleiotropy cause genetic correlation (Lynch & Walsh, 1998). With the low LD in trees, we may expect that pleiotropy is the main reason for the observed adverse genetic correlations between fecundity and growth traits.

However, an adverse correlation may or may not affect a two‐trait trade‐off unless one of traits is related to fitness. Two‐trait trade‐off indicates that fitness cannot be maximized because of competing demands on the tree that share a limiting resource. It may create a challenge for simultaneously improving growth and fecundity with the observed genetic correlation. A trait such as growth does not stabilize at extreme values in the wild, because increases in the allele frequencies of favourable alleles may have negative effects on fitness.

In summary, the consistent negative genetic correlation observed between growth, represented by DBH, and fecundity, represented by CONE, in this large breeding experiment may indicate several concerns:

1. there may be a functional or an evolutionary constraint imposed on the simultaneous improvement of a production trait such as growth and a fitness trait such as fecundity in radiata pine;
2. artificial selection for growth only without considering fecundity will break this evolutionary constraint, and under long‐term recurrent selection and breeding, fitness could be compromised; hence, fecundity should either be monitored or, alternatively, used as a selection criterion, or included as a breeding objective trait;
3. a negative genetic correlation will be a concern for the deployment population, if seedling deployment is the main tool for mass propagation.

In breeding programmes of maize and other crops, yield gain through breeding is usually accompanied with a decline in biomass production of growth‐related traits such as stem height (Evans, 1993). Forest trees present a contrast to crops, in that our main breeding objective is to increase growth rate for stem size (and not the reproductive component). Imposing a strong selection pressure on a growth trait such as DBH is expected to reduce fecundity in radiata pine due to the observed negative genetic correlation. This has been observed in commercial estate plantings, progeny trials, and seed orchards. Selected, elite trees usually produced flowers, later, and with a lower number of flowers (Fielding, 1960, Wu et al., 2004, and Peter Buxton—Tree Breeding Australia, personal communication). Such a negative genetic correlation has a significant implication for both breeding and evolution of the species. In natural populations, fecundity is the most important trait of fitness that is related to survival and maintenance of the population. There must be a balance in natural populations between growth rate and fecundity (a functional or evolutionary constraint). With such a constraint, natural selection will unlikely select a radiata pine tree for faster growth if it reduces its fitness‐related fecundity. Our study is a contrast with a recent review that plant biomass is a reliable estimate of plant fitness (Younginger et al., 2017). But these authors also indicated if larger individuals have higher fecundity and a greater potential to leave viable offspring, it would seem that plant size in successive generations would continue to increase until physical limits were attained. However, there is little empirical evidence for this occurring in nature. In a dozen of tree species cited by the paper, it was observed that their conclusion was only based on phenotypic relationships. As we demonstrated in this study, phenotypic correlation cannot be used to deduce possible evolutionary constraints because phenotypic correlation is influenced by the environmental correlation that is not heritable. We believe it is the genetic correlation that drives the functional or evolutional constraints between growth and fecundity.

However, in forest tree plantations maintaining the balance between growth and fecundity may not be immediately required because the replacement of the old plantation is achieved by replanting of a new tree crop, and not by natural reproduction within the in situ population. The seeds are either from seed orchards using either controlled‐ or open‐pollinated (or assisted mass pollination). However, tree breeding aiming for growth traits only may interrupt the evolutionary constraint, that balance growth rate and fecundity, maintained in natural populations. By interrupting this evolutionary constraint, therefore, there would be a cost in long‐term adaptation of the domesticated tree species. Radiata pine plantations outside the native range not only comprise the commercial planting estate, but also comprise ex situ conservation stands (e.g. approximately 100 provenance plantations derived from the native population collection (Eldridge, 1997; Roger, 2002)). This has added a need to maintain the balance between growth and the fecundity in renewing the plantation and the provenance plantations for the next generation.

Then, what is the best strategy for dealing with such a negative genetic correlation in the breeding programme for radiata pine (Wu & Sanchez, 2011)? Our previous simulation of selection for two negatively correlated traits due to antagonistic pleiotropic gene effects indicate that (1) for short‐term strategy, selection based on two traits simultaneously is an effective strategy for maintaining the value of the correlated secondary trait (such as fecundity) while increasing the value of the primary trait (such as DBH); and (2) for long‐term strategy, dissecting the genetic basis of traits using a large, genome‐wide association study is recommended. This is because selection favouring one trait will cost (decrease) the value of the correlated traits if the correlation is due to pleiotropic loci with antagonistic effects only. Genetic gains for adversely correlated traits (such as DBH and fecundity) could be made for many generations with selective breeding if there are independent loci for each individual trait (besides pleiotropic loci with antagonistic effects for the correlated traits).

## 6 SELECTION STRATEGY TO BALANCE GROWTH TRAIT DBH AND MAINTAIN FECUNDITY IN SHORT‐TERM

**Figure 4:**
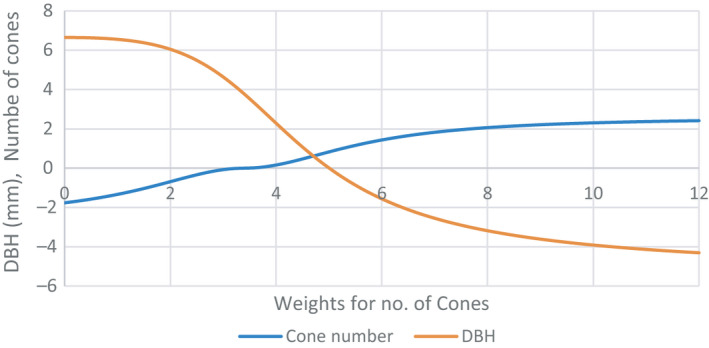
Genetic gain in DBH and CONE (vertical axis) when economic weight for the CONE varies from 0 to 12 relative to DBH (horizontal axis)

Without an understanding of the genetic basis of the negative correlation between traits at the gene level at this stage, we only can use population parameters to develop an optimal selection strategy to balance growth and fecundity. Selection for DBH alone would have resulted in positive genetic gains of 6.65 mm (3.4%) but a negative gain in CONE of 1.76 (19.6%) under selection intensity of 1%. If we maintain CONE (zero change), the genetic gain for DBH would be reduced to 3.76 mm (1.9%). Figure 4 depicts the genetic gain (loss) by changing the economic weight for CONE from 0 to 12 relative to DBH. It can be seen that gains in both are possible from the relative weight of 3.6 to 5 for CONE with the cross‐line point at about 0.6 mm gain for DBH and 0.6 CONE gain under the relative weight of 4.7. Low fecundity in several conifer species (such as Norway spruce (*Picea abies*)) and reduced flowering in advanced generations of conifer breeding (such as radiata pine) are a large concern for tree breeding programmes. In radiata pine, it was observed that flowering was delayed and the numbers of flowers were reduced after mating of related trees (Wu et al., 2004). In Norway spruce, delayed flowering and infrequent flowering is a main constraint to accelerating the breeding programme (Almqvist et al., 2001). Therefore, studies into the inheritance of flowering and its genetic relationship with tree growth (an important breeding objective trait) at the gene level are urgently required. The simultaneous genetic improvement of DBH and CONE is possible even if a negative genetic relationship exists, provided there are independent causal loci for the different traits (Wu & Sanchez, 2011). A possible strategy to maintain fecundity is to use restricted selection strategy at this point in time.

## 7 CONCLUSION

Fecundity as measured by precocity and abundance of female cones is highly heritable in radiata pine with a moderate to high heritability about 0.39–0.61. Fecundity had a lower genetic variation and genotype by site interactions relative to DBH, based on this study. With such high heritability, fecundity can be increased or reduced genetically by selective breeding.

Site had significant impact on fecundity, mainly due to the amount of rainfall of the tested sites in this experiment. Drier sites had higher fecundity.

Moderate but negative genetic correlations between fecundity and growth (average of −0.39) were observed in this large sample of the Australiasian radiata pine population. This indicates that there is an evolutionary constraint in natural populations and ex situ conservation populations that imposes stabilizing selection of growth and fecundity. Selective breeding to improve tree growth rate alone could reduce fecundity of radiata pine (and *vice versa)*. However, a selection strategy for increasing wood production while maintaining acceptable fecundity should be developed for short‐ and long‐term breeding programme. Fecundity should be included as an objective trait in long‐term breeding programmes and in ex situ conservation stands, such as radiata pine.

In the short term, a constrained selection index approach is recommended to maintain the fecundity level while maximizing genetic gain for growth rate. For long‐term breeding strategy and ex situ conservation, dissecting the genetic basis of the negatively correlated traits at the gene level is required.

## Data Availability

The data that support the findings of this study are available from Tree Breeding Australia (http://www.stba.com.au/). Restrictions apply to the availability of these data, which were used under license for this study. Data are available from the authors with the permission of Tree Breeding Australia.
